# Health status and social determinants among Roma and Sinti communities in Italy: a cross-sectional study with a focus on sex-based inequalities

**DOI:** 10.3389/fsoc.2026.1717075

**Published:** 2026-04-30

**Authors:** Davide Pata, Danilo Buonsenso, Bettina Camara, Maria Luisa Ricciardi, Alessandro Solipaca, Alessia D'Errico, Francesca Colaiaco, Umberto Moscato, Walter Malorni

**Affiliations:** 1 Center for Global Health Research and Studies, Istituto di Igiene, Catholic University of the Sacred Heart, Rome, Italy; 2 Department of Woman and Child Health and Public Health, Fondazione Policlinico Universitario A. Gemelli IRCCS, Rome, Italy; 3 Medicine and Surgery, Catholic University of the Sacred Heart, Rome, Italy; 4 National Observatory on Health in the Italian Regions, Catholic University of the Sacred Heart, Rome, Italy; 5 Istituto Nazionale di Statistica-ISTAT, Rome, Italy; 6 Italian Red Cross Association, Rome, Italy

**Keywords:** access to cures, chronic diseases, disease prevention, inequalities, minorities, public health, Roma, Sinti

## Abstract

**Background:**

This study investigates the health status and social determinants of health among Roma and Sinti communities residing in Italy living in settlements and camps, with a particular focus on education, housing conditions, and particular attention to sex differences and inequalities.

**Methods:**

A cross-sectional survey was conducted on 619 individuals, using both quantitative and qualitative methodologies to assess demographic characteristics, lifestyle behaviors, preventive healthcare practices, vaccination history, and housing conditions.

**Results:**

The sample consisted of 51.4% women and 48.6% men, with the majority being Italian citizens (69.5%). Findings revealed significant disparities compared to the general Italian population. Education and employment levels were markedly lower, especially among women, and early marriage and labor participation were common. Lifestyle behaviors showed pronounced differences between male and female participants, with men more likely to smoke and consume alcohol, while women reported poorer perceived health. Preventive health practices, including vaccinations and screenings, were consistently lower than national averages, though school attendance was associated with higher vaccination coverage. Housing deprivation was prevalent but showed limited direct statistical association with health status, although it correlated with lower adherence to preventive screenings.

**Conclusions:**

The results underscore the intersectional vulnerabilities of Roma and Sinti populations, highlighting the roles of sex, education, and systemic barriers in shaping health outcomes. While the survey's limitations prevent statistical generalization, the findings provide critical insights into health inequalities and suggest that targeted interventions in education, housing, and healthcare access are essential to improve the wellbeing of these marginalized communities.

## 1 Introduction

The Romani—also known as Roma—are an ethnic group that resides mostly across eastern and southern Europe, most often making up a small minority group (Hajioff and McKee, 2000). The Sinti people are a subgroup of the Roma, an inherently heterogeneous group with varying cultural and social practices (Kóczé and Rövid, 2017a). The Council of Europe estimates only around 0.25% of the Italian population is composed of Roma and Sinti individuals, somewhere between 120,000 and 180,000 people (Council of Europe, 2021). Despite common misconception, about 50% of those individuals hold an Italian citizenship, and only an estimated 3% are nomadic (Sigona and Monasta, 2006). A majority of the Roma and Sinti population residing in Italy are young, with 60% of them being of pediatric age [18 years of age or less; European Union Agency for Fundamental Rights (FRA), 2016]. Recent reports have suggested that, despite relatively low numbers of Romani residents in comparison to other European countries, Italy has one of the highest levels of reported discriminatory and racially biased sentiment against the ethnic group (Cook et al., 2013). Housing scarcity for Roma and Sinti groups is highest in Italy, where there is still a continued establishment of policies that add to roadblocks to the health, safety and quality of life of individuals (Monasta et al., 2012a).

Discrimination against the Roma and Sinti people has been prevalent across Europe for centuries, in part due to their nomadic history and misconceptions about their cultural norms (Liegeois, 1994). Following the events of World War II and the persecution of Romani populations during the holocaust, sentiment against individuals saw little improvement (Lewy, 2000). New groups of migrants arriving after the war and the turn of the century experienced ongoing social and economic isolation, residing in the outskirts of society and facing barriers to housing, education and employment (McGarry, 2017). Numerous efforts by Europe-wide organizations aimed to establish policies and social programs with the hopes of improving the integration and quality of life of Romani populations across the continent (European Commission, 2011). Nonetheless, precarious conditions and reduced access make the Roma and Sinti community particularly vulnerable to worse health outcomes than other ethnic minorities, and therefore less likely to experience improved socio-economic status with each generation (Vokó et al., 2009). These factors ultimately set the community apart from ethnically Italian nationals, and exacerbate social and economic disparity (Foldes and Covaci, 2012).

The objective of this manuscript was to gather and analyze data from Roma and Sinti individuals residing in Italy, in order to establish a foundational understanding of the health status of this ethnic group in comparison to other Italians. The present study is conceptually informed by the framework of the social determinants of health, as defined by the WHO Commission on Social Determinants of Health. According to this perspective, health inequalities arise from the interaction between structural determinants (such as socioeconomic and political context, discrimination, and social stratification) and intermediary determinants including living conditions, education, employment, and access to healthcare. Roma and Sinti populations in Europe have historically experienced structural exclusion across multiple domains, including housing, education, and labor markets. These structural disadvantages may translate into differential exposure to health risks and unequal access to preventive services and healthcare resources. In this study, we therefore explore several social determinants that may influence health outcomes within Roma and Sinti communities living in Italy, including education, employment status, housing conditions, and access to preventive healthcare services. This analysis aims to evaluate the factors that may contribute to the wellbeing of the Romani minorities, and create a direction for future investigation, as well as pathways for improvement for the future of the community as a whole.

## 2 Methods

### 2.1 Data source and study samples

Studies on Roma and Sinti health and barriers to access to the healthcare system have some important shortcomings, particularly in relation to the health status and access to care for marginalized minorities. The survey intends to systematize what is known in the literature by providing information on the health status and access to the National Health System (NHS). Furthermore, it intends to delve into the barriers encountered by the Roma and Sinti communities using quantitative and qualitative methodologies. This study will aim to guide the development of the other phases of this same project but also to plan, in the medium-long term, measures to implement to reduce the health disparities by allowing the focus on specific targets and territories. In particular, the development of this activity will focus on the definition and implementation of a survey on the health status of Romani communities living in settlements and camps in Italy and on barriers to access to the NHS. It will have a specific focus on the situation of children and adolescents and other vulnerable people, including women, and will use validated quantitative and qualitative methodologies to investigate perceived health status. The questionnaire development process required the involvement of researchers from the Center for Global Health at the Catholic University of Rome, collaboration with Italian Red Cross volunteers familiar with the Roma and Sinti communities, the use of previously validated survey items commonly used in public health research. The questionnaires used are available in the Supplementary Files 1–3.

Participants were eligible for inclusion if they were members of Roma or Sinti communities living in settlements or camps in Italy and were aged 18 years or older at the time of the survey. Although the broader project included separate questionnaires targeting children, adolescents, and adult women, the analyses presented in this article are restricted to adult respondents who completed the adult questionnaire. Therefore, all results reported in this manuscript refer exclusively to individuals aged 18 years and above.

The survey, developed by the staff of the Global Health Center of the Catholic University of Rome, was distributed among the Roma and Sinti community by the research group through volunteers of the Italian Red Cross Association (ItRC).

To identify the ItRC local branches best suited to contribute to the data collection activities targeting the project's population, a Call for Expressions of Interest was launched. This initiative involved the entire national ItRC network—comprising approximately 730 local branches—with the aim of mapping the presence of Roma and Sinti groups and assessing the quality of ongoing relationships and interventions carried out by the branches with these communities.

Local branches were selected over a 2-month period through an online questionnaire focusing on the following key aspects:

- Existence of services offered to communities;
- Type of services most provided (social, socio-health);
- Locations and delivery methods of services;
- Number of volunteers involved;
- Estimated number of beneficiaries reached;
- Number of settlements served.

The collected data enabled the identification of 39 ItRC local branches actively engaged in delivering services to RS populations, with a potential reach of 44 settlements across the national territory. During the subsequent engagement phase, the selected branches participated in virtual co-design sessions aimed at collaboratively developing effective methodologies for administering the health questionnaire. These meetings also served to align project objectives, explore the specific characteristics of local RS communities, and jointly address operational challenges. As a result of this engagement phase, 26 ItRC branches formally confirmed their participation, while 8 later withdrew due to the relocation of RS groups, mistrust or reluctance from the target population, and external events (e.g., police-led evictions, fires). The final selection designated a focal point for each local branch. In this role, these facilitators act as crucial intermediaries who foster and support interaction between volunteers and members of the participating communities. Their facilitation emphasizes engaging community members as active partners in the co-design and co-planning of project activities and objectives, thereby enhancing the effectiveness and cultural appropriateness of project interventions. The selection criteria prioritized territorial distribution and the existence of established trust-based relationships with participants. As a result of the data collection activities, 18 ItRC local branches collected a total of 619 completed questionnaires.

Participants were recruited through the ItRC local branches working in Roma and Sinti settlements. Volunteers and community mediators approached potential participants during routine outreach activities and invited them to participate in the survey. Given the linguistic diversity present within Roma and Sinti communities, questionnaires were administered with the support of community mediators familiar with the local context. When necessary, explanations were provided in languages understood by participants to ensure comprehension of the questions. The recruitment procedure therefore relied on convenience sampling rather than probabilistic sampling. Selection occurred at the individual level, although interviews often took place within households or shared community spaces. Participation was voluntary and no incentives were provided.

Because recruitment was facilitated through ItRC branches that had pre-existing relationships with local communities, the sample should not be considered statistically representative of the Roma and Sinti population living in Italy.

### 2.2 Measures of interest

Individuals were interviewed regarding their age, biological sex (male/female), educational and professional background. Lifestyle questions such as frequency of sports, smoking and alcohol consumption were also posed. Participants were asked about their perceived health status, number of preventative health tests, vaccination history.

The survey collected information on biological sex (male/female) as self-reported by participants. In the analysis, differences between male and female respondents are interpreted in relation to gender roles and social norms, which may influence health behaviors, opportunities, and access to resources within Roma and Sinti communities.

Perceived health status was assessed using a self-rated health question (“How would you describe your current health status?”) with response options ranging from “very good,” “good,” “fair,” “poor,” to “very poor.”

Preventive screening variables (cholesterol testing, blood glucose testing, fecal occult blood test, Pap smear, and mammography) were collected through self-report. Participants were asked whether they had ever undergone these tests and, when applicable, when the test was performed.

Vaccination history was also inquired, including Measles, Mumps, Rubella, Influenza, Pneumonia, Tuberculosis, Tetanus, Diphtheria, Pertussis, Polio, Chickenpox, Hepatitis B, and COVID. Vaccination status in this study refers to self-reported receipt of at least one vaccine among the list of vaccines included in the questionnaire.

Housing hardship is a theoretical construct that cannot be measured directly. The survey identified several characteristics of dwellings that can describe housing hardship; these characteristics are qualitative in nature and cannot be ordered. In particular, the survey collected variables describing housing conditions, including dwelling size, water supply, gas connection, heating system, number of cohabitants, number of windows, availability of internet connection, presence of a washing machine and freezer, and access to waste collection services. Housing deprivation was assessed through a composite indicator constructed using Multiple Correspondence Analysis (MCA). Multiple Correspondence Analysis (MCA) is a technique applied to qualitative data that makes it possible to identify orthogonal factors capable of approximating the latent dimension of the phenomenon of “housing hardship.” It is a non-parametric methodology that extracts a subspace through a constrained optimization technique, minimizing the loss of information. The composite indicator was represented by categorized into four levels, assigned on the basis of the quartiles of the units' factor scores on the first factorial axis, representing increasing levels of housing deprivation: low, medium-low, medium-high, and high. The first factorial axis explains 61% of the inertia.

Descriptive statistics were used to summarize the characteristics of the study population. Categorical variables are reported as absolute numbers and percentages.

Associations between categorical variables were assessed using Fisher's exact test when expected cell counts were small. For contingency tables with larger sample sizes, Pearson's chi-square test was applied. Statistical significance was set at *p* < 0.05.

Given the exploratory nature of the study and the presence of missing values in some variables, analyses were primarily descriptive and bivariate. Multivariable models were not performed and this limitation is discussed in the limitations section.

## 3 Results

### 3.1 Demographic structure

A total of 619 individuals were included in the analysis, of whom 51.4% were women (*n* = 318) and 48.6% were men (*n* = 301). The majority of participants were younger than 54 years (women: 78.9%, *n* = 251/318; men: 74.1%, *n* = 223/301). The most represented age group was 18–23 years among men (15.0%, *n* = 45/301) and 34–38 years among women (14.1%, *n* = 45/318). Most participants were Italian citizens (70.1%, *n* = 434), followed by foreign nationals (28.6%, *n* = 177) and stateless individuals (1.3%, *n* = 8). Among respondents, 77.4% (*n* = 479) identified as Roma/Sinti. Regarding marital status, 49.3% (*n* = 305) were married, 37.7% (*n* = 233) single, 8.1% (*n* = 50) widowed, and 5.0% (*n* = 31) separated or divorced. Table 1 summarizes the demographic structure of the population included in the study.

**Table 1:** Sample characteristics.

Variable	Category	Total *n* (%)	Men *n* (%)	Women *n* (%)
Sex	Men	301 (48.6%)	—	—
	Women	318 (51.4%)	—	—
Age < 54	Yes	474 (76.6%)	223 (74.1%)	251 (78.9%)
Most represented age group	Men (18–23)	45 (15.0%)	—	—
	Women (34–38)	45 (14.1%)	—	—
Citizenship	Italian	434 (70.1%)	200	212
	Foreign	177 (28.6%)	84	93
	Stateless	8 (1.3%)	3	5
Ethnicity	Roma/Sinti	479 (77.4%)	—	—
	Other	140 (22.6%)	—	—
Marital status	Married	305 (49.3%)	—	—
	Single	233 (37.7%)	—	—
	Widowed	50 (8.1%)	—	—
	Separated/divorced	31 (5.0%)	—	—

### 3.2 Education and employment status

**Figure 1:**
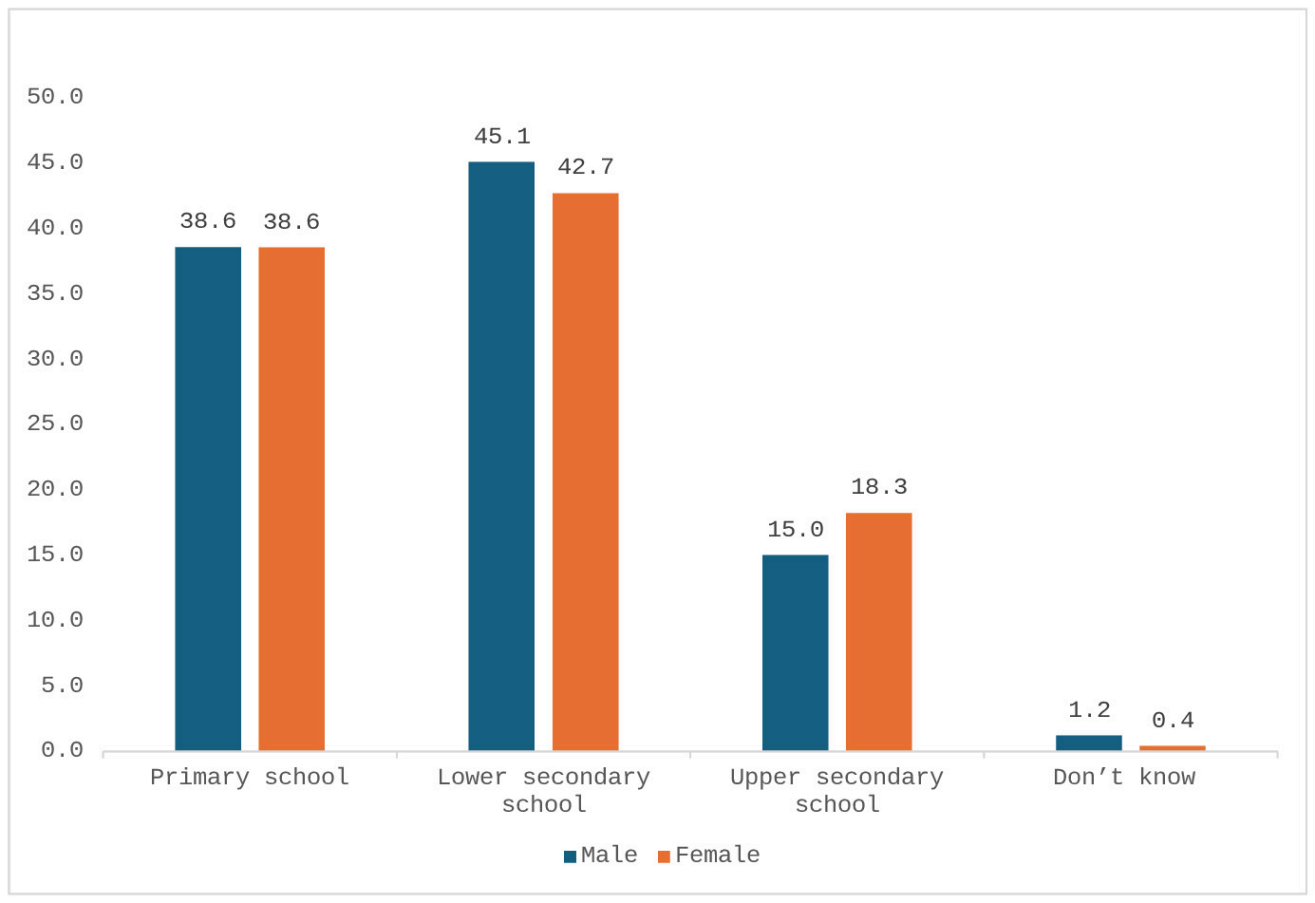
Distribution by level of school attended (percentage values).

**Figure 2:**
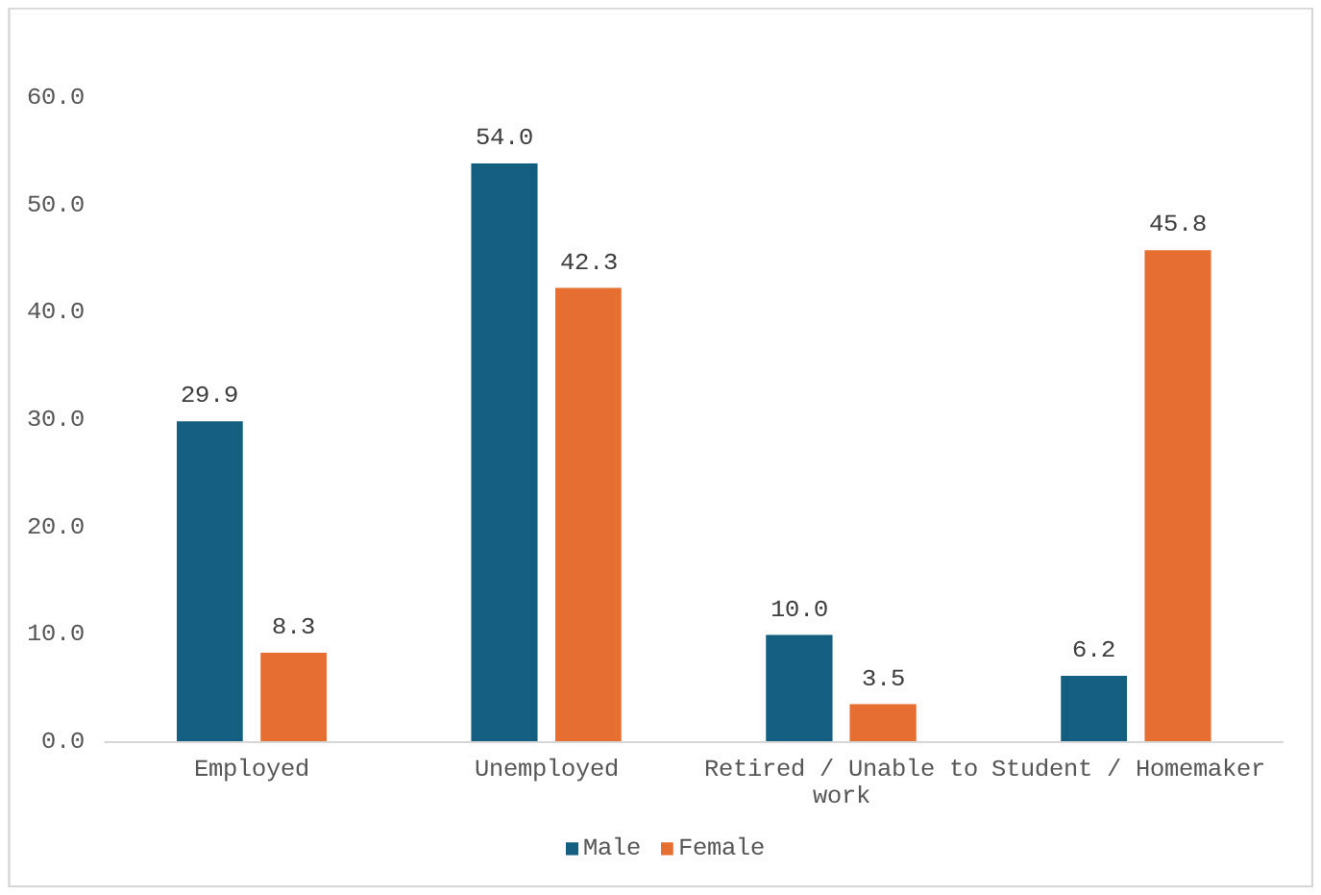
Professional status by gender (percentage values).

School attendance was reported by 82.6% of men (*n* = 249/301) and 76.7% of women (*n* = 244/318), with no statistically significant difference (95% CI: −0.3 to 12.1; *p* = 0.0728, Figure 1). High school attainment was reported by 15.0% of men (*n* = 45/301) and 18.3% of women (*n* = 58/318), with no significant difference (95% CI: −2.3 to 8.9; *p* = 0.6127). Among individuals who did not attend school (*n* = 126), 58.5% (*n* = 74) reported being unable to read in Italian, 32.5% (*n* = 41) reported major difficulties, and only 7.1% (*n* = 9) reported sufficient or higher reading ability. Employment status differed significantly between sexes. Among men, 29.9% (*n* = 90/301) were employed and 44.7% (*n* = 135/301) were seeking employment. In contrast, 8.3% of women (*n* = 26/318, Figure 2) were employed and 32.7% (*n* = 104/318) were seeking employment. Additionally, 10.0% of men (*n* = 30/301) and 3.5% of women (*n* = 11/318) were retired or unfit for work. The difference in employment status between sexes was statistically significant (95% CI: 15.7–27.7; *p* < 0.0001). Table 2 lists the educational and employment status of the population included in the study.

**Table 2:** Education and employment.

Variable	Category	Men *n* (%)	Women *n* (%)	95% CI	*p*-value
School attendance	Yes	249 (82.6%)	244 (76.7%)	−0.3–12.1	0.0728
	No	52 (17.4%)	74 (23.3%)	—	—
High school	Yes	45 (15.0%)	58 (18.3%)	−2.3–8.9	0.6127
	No	256 (85.0%)	260 (81.7%)	—	—
Literacy (no school, *n* = 126)	Cannot read	74 (58.5%)	—	—	—
	Difficulty reading	41 (32.5%)	—	—	—
	Adequate reading	9 (7.1%)	—	—	—
Employment	Employed	90 (29.9%)	26 (8.3%)	15.7–27.7	< 0.0001
	Seeking job	135 (44.7%)	104 (32.7%)	—	—
	Retired/unfit	30 (10.0%)	11 (3.5%)	—	—

### 3.3 Wellbeing and lifestyle

**Figure 3:**
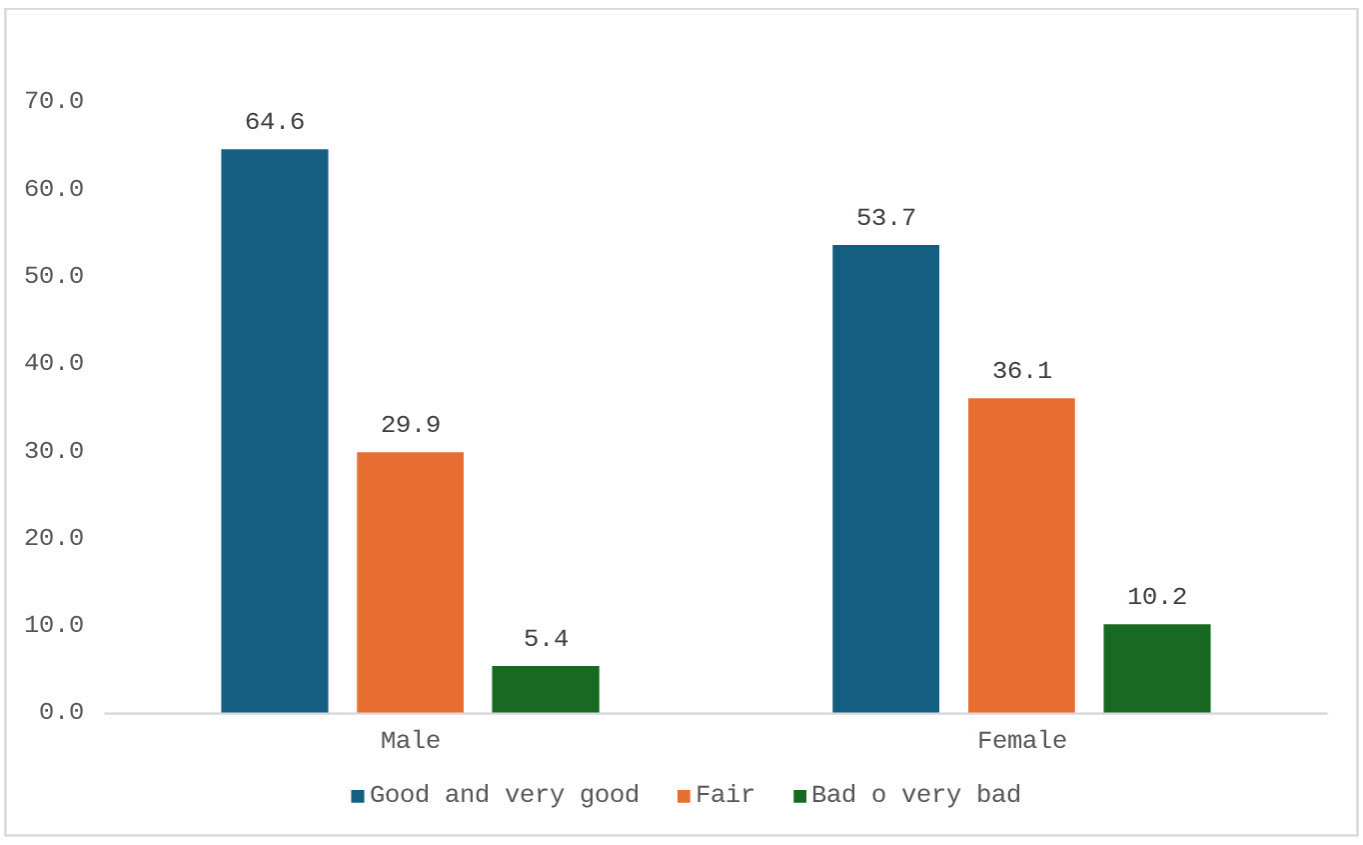
Perceived health status by gender (percentage values).

**Figure 4:**
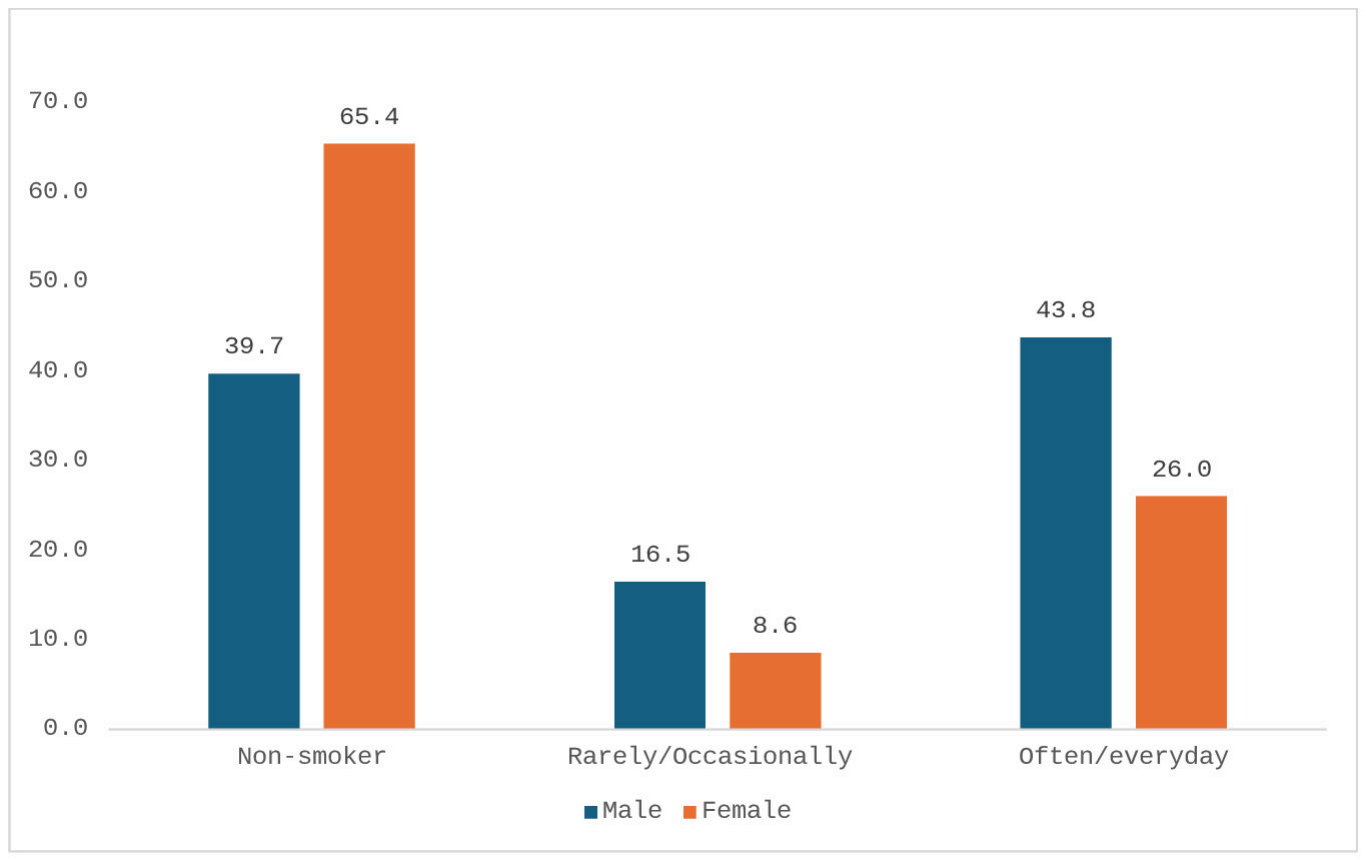
Smokers by gender (percentage values).

**Figure 5:**
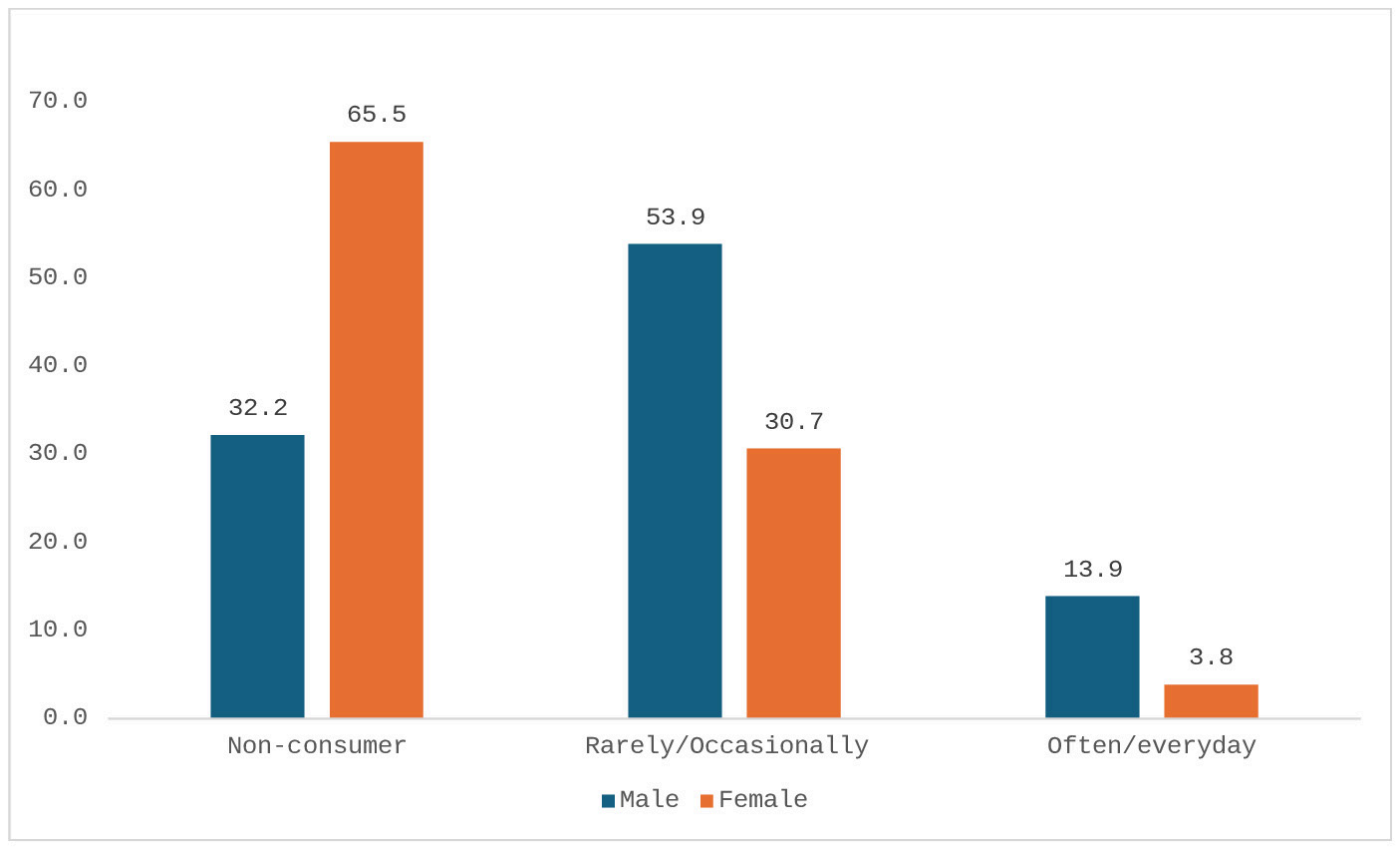
Alcohol consumers by gender (percentage values).

**Figure 6:**
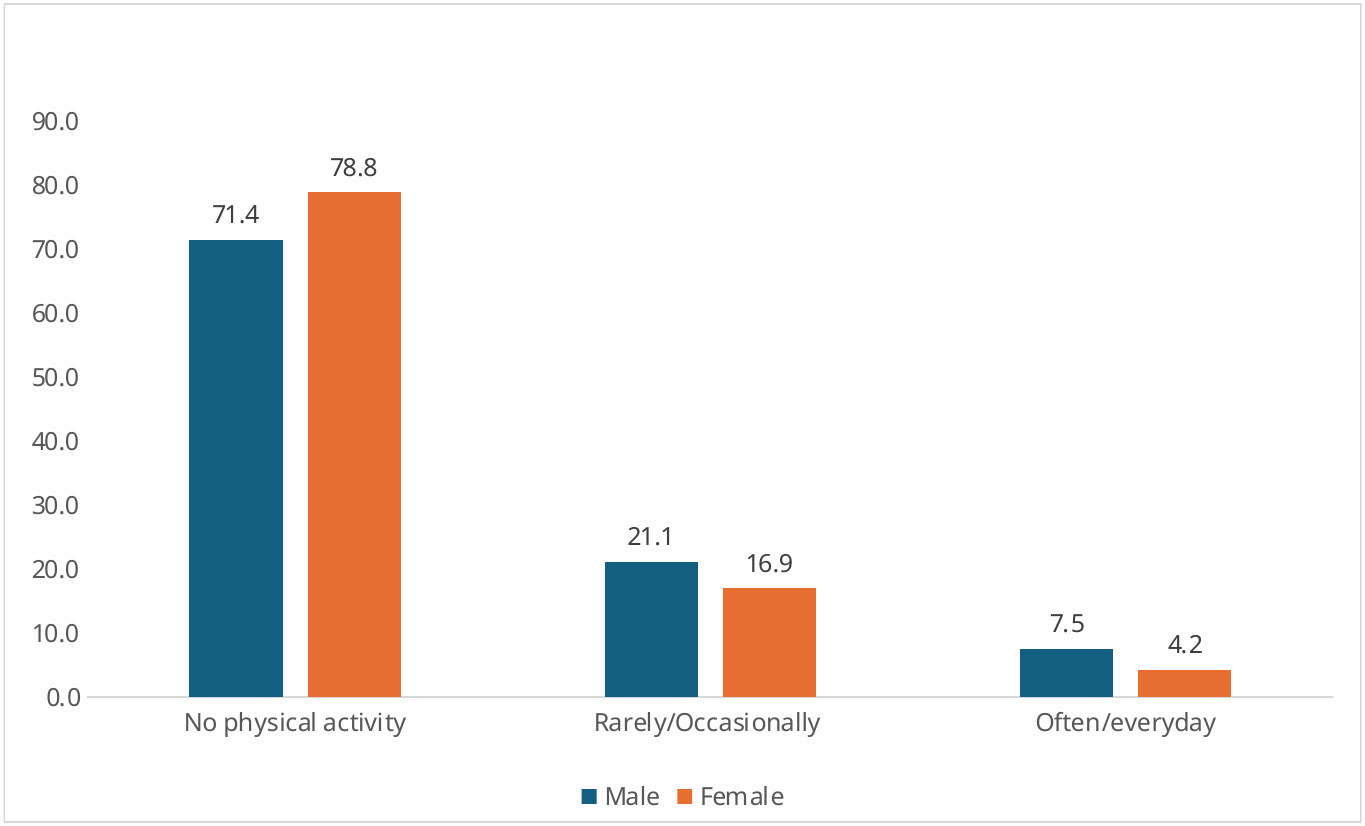
Physical activity by gender (percentage values).

Perceived good or very good health was reported by 64.6% of men (*n* = 194/301) and 53.7% of women (*n* = 182/318), while poor or very poor health was reported by 5.4% (*n* = 16/301) of men and 10.2% (*n* = 35/318) of women (Figure 3). Smoking was significantly more frequent among men (43.8%, *n* = 132/301) than women (26.0%, *n* = 83/318) (95% CI: 10.7–24.9; *p* < 0.0001). Alcohol consumption was also higher among men (13.9%, *n* = 42/301) compared to women (3.8%, *n* = 12/318) (95% CI: 5.2–15.0; *p* < 0.0001, Figures 4, 5). Regular physical activity was low, reported by 7.5% of men (*n* = 23/301) and 4.2% of women (*n* = 13/318), while 71.4% of men (*n* = 215/301) and 78.8% of women (*n* = 251/318) reported not practicing any physical activity (95% CI: −0.6 to 7.4; *p* = 0.0755, Figure 6). Table 3 summarizes lifestyle and perceived health of the population.

**Table 3:** Lifestyle and perceived health.

Variable	Category	Men *n* (%)	Women *n* (%)	95% CI	*p*-value
Perceived health	Good/very good	194 (64.6%)	182 (53.7%)	—	—
	Poor/very poor	16 (5.4%)	35 (10.2%)	—	—
Smoking	Yes	132 (43.8%)	83 (26.0%)	10.7–24.9	< 0.0001
Alcohol	Yes	42 (13.9%)	12 (3.8%)	5.2–15.0	< 0.0001
Physical activity	Regular	23 (7.5%)	13 (4.2%)	−0.6–7.4	0.0755
	None	215 (71.4%)	251 (78.8%)	—	—

### 3.4 Disease prevention

**Figure 7:**
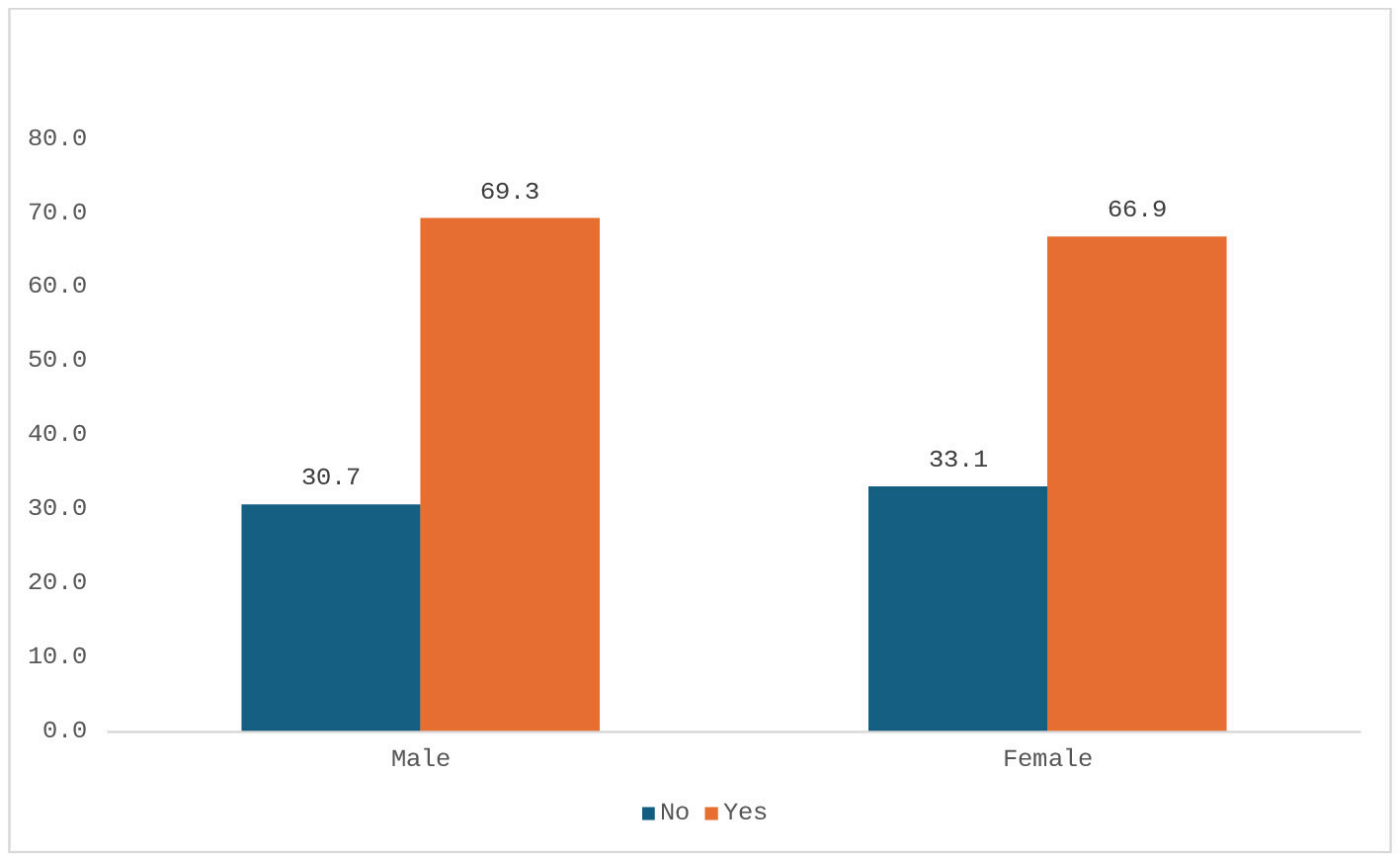
Individuals receiving at least one vaccination by gender (percentage values).

**Figure 8:**
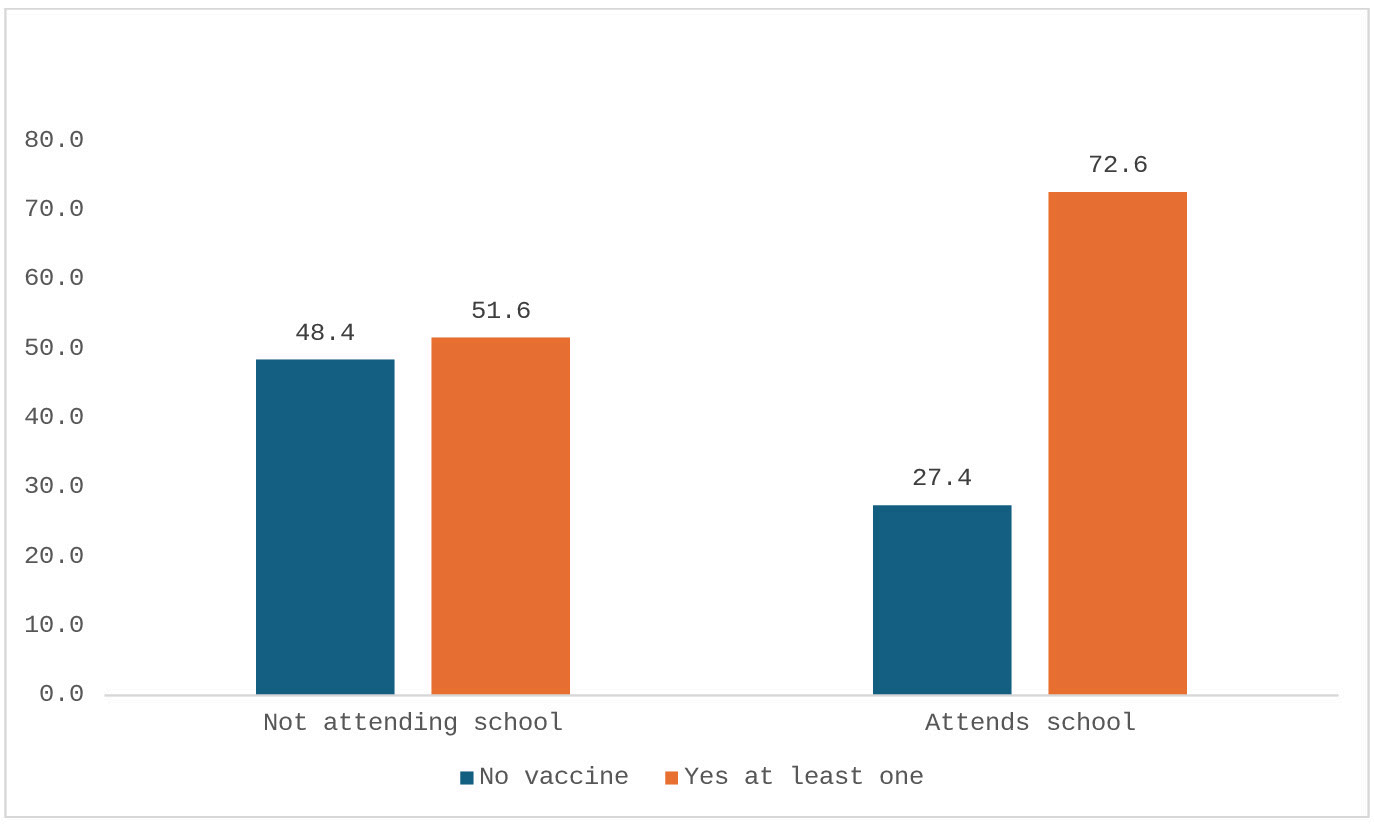
Individuals receiving at least one vaccination by school attendance (percentage values).

Vaccination coverage did not differ significantly between sexes, with 69.3% of men (*n* = 208/301) and 66.9% of women (*n* = 213/318) reporting at least one vaccination (95% CI: −5.1 to 9.9; *p* = 0.5457, Figure 7). Vaccination rates were significantly higher among individuals who had attended school (72.6%, *n* = 358/493) compared to those who had not (51.6%, *n* = 65/126) (95% CI: 13–29; *p* < 0.0001) (Figure 8).

Cholesterol testing had been performed in 31.6% of men (*n* = 95/301) and 35.4% of women (*n* = 112/318). However, 41.5% of men (*n* = 125/301) and 32.5% of women (*n* = 103/318) reported never having undergone the test (95% CI: −10.2 to 2.6; *p* = 0.0873, Supplementary material).

Blood glucose testing was performed annually by 31.3% of men (*n* = 94/301) and 36.2% of women (*n* = 115/318), while 40.6% of men (*n* = 122/301) and 33.6% of women (*n* = 107/318) reported never having undergone the test (95% CI: −11.5 to 1.7; *p* = 0.3747).

Fecal occult blood testing was reported by 7.1% of men (*n* = 21/301) and 8.5% of women (*n* = 27/318).

Among women, 31.6% (*n* = 100/318) reported having undergone a Pap test, while 23.9% (*n* = 76/318) reported having undergone mammography. These differences were not statistically significant (Pap test: *p* = 0.6167; mammography: *p* = 0.9761). Table 4 lists vaccination and screening tests performed.

**Table 4:** Preventive healthcare and screening.

Variable	Category	Men *n* (%)	Women *n* (%)	95% CI	*p*-value
Vaccination (≥1)	Yes	208 (69.3%)	213 (66.9%)	−5.1 to 9.9	0.5457
Cholesterol test	Yes	95 (31.6%)	112 (35.4%)	−10.2 to 2.6	0.0873
	Never	125 (41.5%)	103 (32.5%)	—	—
Blood glucose	Annual	94 (31.3%)	115 (36.2%)	−11.5 to 1.7	0.3747
	Never	122 (40.6%)	107 (33.6%)	—	—
Fecal occult blood test	Yes	21 (7.1%)	27 (8.5%)	—	—
Pap test	Yes	—	100 (31.6%)	—	0.6167
Mammography	Yes	—	76 (23.9%)	—	0.9761

### 3.5 Housing

Housing deprivation affected 26.7% of men (*n* = 80/301) and 22.1% of women (*n* = 70/318), with no statistically significant difference (95% CI: −1.1 to 9.5; *p* = 0.1822). A significant association was observed between housing deprivation and citizenship (*p* = 0.001, Supplementary material), with higher prevalence among foreign participants (36.2%, *n* = 64/177) compared to Italian citizens (19.1%, *n* = 83/434) and stateless individuals (25.0%, *n* = 2/8). No statistically significant association was found between housing deprivation and perceived health status, although a higher proportion of individuals with high deprivation reported poor health (8.4% vs. 6.6%, Supplementary material). Table 5 reports the housing conditions detected.

**Table 5:** Housing conditions.

Variable	Category	Men *n* (%)	Women *n* (%)	95% CI	*p*-value
Housing deprivation	Yes	80 (26.7%)	70 (22.1%)	−1.1 to 9.5	0.1822
Citizenship	Italian	83 (19.1%)	—	—	0.001
	Foreign	64 (36.2%)	—	—	—
	Stateless	2 (25.0%)	—	—	—

## 4 Discussion

The data obtained through the interviews suggest a substantial contrast between the general Italian population and Roma and Sinti communities across several dimensions of quality of life, including education, employment, health behaviors, and access to preventive healthcare services.

Although the present sample is not statistically representative of Roma and Sinti populations living in Italy, the findings provide relevant descriptive insights when interpreted alongside national statistics. At the same time, it is important to acknowledge that Roma and Sinti populations are highly heterogeneous, with differences in migration history, socioeconomic conditions, and levels of integration. The present study does not allow for a detailed exploration of this internal diversity, which represents an important direction for future research.

Since the attempted establishment of policies aimed at reducing discrimination in the late 1960s (Liégeois, 2013), continued systemic efforts have been put in place (European Commission, 2020), though it seems little to no change in the quality of life of the Romani communities has been seen compared to other European ethnicities (Nanda, 2022). In 2012, Monasta et al. conducted a review of studies into health conditions of Roma and Sinti in Italy, and found a lack in prevalent data into the social determinants of health and disease affecting the community (Monasta et al., 2012b). The lack of relevant and robust data places obstacles in the path toward improved health and wellbeing of individuals, and prolongs the existence of preventable health struggles. This study aimed to evaluate the intersectionalities of perceived health status of the community, and provide an updated outlook on Romani individuals in comparison to Italian residents of other ethnic backgrounds.

### 4.1 Sex-based differences

Gender and family roles are, notably, very established and unlikely to be strayed from in Roma and Sinti communities (Afanasieva et al., 2020).

The analyses identified several differences between male and female participants. Women reported lower employment rates and poorer perceived health, whereas men reported higher prevalence of smoking and alcohol consumption. These findings are consistent with patterns observed in other socioeconomically disadvantaged populations.

It is important to clarify that the present study collected data on biological sex (male/female), and therefore the observed differences should be interpreted as sex-based differences. The study did not include direct measures of gender-related constructs such as roles, norms, or identities.

However, the literature suggests that differences in health behaviors and access to resources are often influenced by social and cultural expectations. In many Roma and Sinti contexts, family organization and social participation may follow structured patterns, which can influence educational trajectories, employment opportunities, and health-related behaviors. While these aspects were not directly measured, they may provide a contextual framework for interpreting the observed differences.

In many Roma contexts, traditional sex expectations continue to structure family organization and social participation, often assigning caregiving and domestic responsibilities to women while men are expected to assume the role of economic providers. These social norms may influence educational trajectories, employment opportunities, and patterns of health behavior. Compared to general Italian society, that still is affected by traditional sentiments and beliefs, the gaps in sex equality in the Romani is still stark and greatly impacts the social and economic structures of the community (Merhaut, 2019).

Social studies have indicated that men are more likely to partake in unhealthy behavior, particularly in the context of marginalized communities (Thorpe et al., 2015). Alcohol and smoking habits have been extensively studied in terms of their relationship with gender as signifiers of masculinity. Excessive drinking culture has been associated with socialization and masculine behavior amongst men and boys across cultures (de Visser and McDonnell, 2011; Emslie et al., 2013; Iwamoto and Smiler, 2013). Furthermore, tobacco use has been seen to be related to expressions of masculinity, which was noted within traditional heterosexual relationships (White et al., 2012; Kwon et al., 2014), much like the ones seen within Romani communities.

Despite the data showing no significant sex differences between screenings like cholesterol levels, it has been suggested that men are also less likely to perform health checks and health seeking behavior due to fear of being seen as fragile or feminine (Wells et al., 2014). Socially disadvantaged women, however, face more barriers to health screening and therefore are also less likely to pursue preventative medicine (Peters, 2012). Notably, however, the large difference noted between employment and education has been consistently seen in studies spanning cultures and generations. In marginalized communities that exemplify the classic male-provider/female caregiver dynamic, women tend to shoulder more unpaid labor such as housework and child care, while men are more encouraged to seek out employment outside the home (Cislaghi and Heise, 2020). Roma and Sinti youth tend to also marry younger, which can lead to earlier education abandonment (Inel Manav, 2024). Interestingly, women interviewed were less likely to attend school but more likely to reach high school. This could be in part due to boys being pulled out of school to start manual labor (Robayo-Abril et al., 2019), and girls staying at school until marriage, which still tends to end their education and employment journey at a young age (Sakamoto et al., 2022).

In the present study, women reported lower employment rates and poorer perceived health compared to men, while men reported higher levels of smoking and alcohol consumption. These patterns may reflect gendered behavioral expectations that have been documented in several studies of marginalized populations, where masculinity is sometimes associated with risk-related behaviors such as tobacco and alcohol use. At the same time, women may face structural barriers that limit their participation in education and formal employment, with potential consequences for socioeconomic status and health outcomes. Beyond the differences identified in this survey, a growing body of literature has highlighted specific vulnerabilities affecting Roma women across Europe (Kóczé and Rövid, 2017b). These include early marriage and early motherhood, limited access to reproductive healthcare, and exposure to gender-based discrimination and violence. Such factors may contribute to reduced autonomy in health-related decision-making and lower utilization of preventive health services. In addition, structural discrimination affecting Roma populations more broadly may intersect with gender inequalities, producing multiple and overlapping forms of disadvantage for Roma women.

Although the present study did not directly investigate issues such as reproductive health, early motherhood, or gender-based violence, the observed differences in education, employment, and perceived health status between male and female respondents should be interpreted within this broader gendered context. Understanding how sex norms interact with social determinants such as education, housing conditions, and access to healthcare is therefore essential for designing effective public health interventions targeting Roma and Sinti communities.

Future research should further explore gender-specific dimensions of health within these populations, including reproductive health, maternal health, and experiences of gender-based violence, in order to provide a more comprehensive understanding of the mechanisms through which gender inequalities influence health outcomes.

### 4.2 The impact of education

Education seemed to have the greatest impact on preventative measures such as vaccination and female cancer screenings (Supplementary material). Nonetheless, vaccination rates were still low, 69.3% in men and 66.9% in women, when the WHO recommends >90% vaccination coverage for disease prevention amongst populations (WHO, 2025). Missed school can result not only in missed vaccination and dental checks that are organized through the Italian primary school system, but can also lead to children missing free school meals which can provide vital nutrition sources (Giampaolo et al., 2021). As well, education can empower individuals to navigate often complicated appointment seeking and scheduling, and provide reading and numerical skills necessary to understand diagnostic decisions, medicine labels and vaccination leaflets.

Roma and Sinti early school dropout rate reported by participants is significantly higher than that of the average national rate (Vegliante et al., 2024). Poor education is a determinant of poorer health outcomes for all Italian citizens, but the structural and systemic discrimination against the Romani communities causes the risks to be much higher. An estimated 62.7% of Italians complete a high school diploma (Baldazzi, 2021), while less than 20% of Roma men and women reported to have stayed in school until high school (Figure 1). As well, the early entry to the labor market and manual jobs instead of remaining in school could also be implicated in poorer health compared to the general Italian population. The Accumulation of these issues can in part explain why the average life expectancy in Italy is on average 82 years, and 10–12 years shorter for Roma and Sinti.

### 4.3 How housing plays a part

The analyses carried out highlight that housing hardship does not always have a significant effect. In fact, no statistically significant relationships were highlighted between housing hardship and health conditions. However, it is notable that preventative practices are higher in the group of interviewees with repeatedly low housing hardship, in particular for flu vaccinations, pap tests and mammograms. Studies have shown a similar pattern of housing security positively correlating with better health practices (Rolfe et al., 2020). Maqbool and colleagues argued that the lack of appropriate housing can shift priorities away from non-urgent medical appointments, instead placing importance on more immediate needs such as food and shelter (Maqbool et al., 2015). Significantly, housing tends to highly influence school attendance in children (Klein et al., 2020). As discussed, decreased school presence can negatively impact health choices, and therefore be directly implicated in the relationship between housing and community health.

### 4.4 Future directions for better health

The future of Roma and Sinti communities will depend highly on systemic changes and tackling gender issues such as early labor for boys and early marriage for girls. Closing health gaps will require improvements from crowded settlements to proper housing, improved school attendance, better access to healthcare, gender and youth specific programs, and greater participation in policy and decision making. This study provides a minimal outlook into the intricate role of social determinants of health. However, the analyses presented allow for some primary understanding of avenues for improvement and how the future of this community depends on a variety of factors, beyond those discussed in this study. Our findings provide descriptive insights into social determinants of health but cannot be generalized to the entire Roma and Sinti population in Italy.

### 4.5 Study limitation

This study has several limitations. First, the sample was obtained through convenience sampling and therefore cannot be considered statistically representative of the Roma and Sinti populations living in Italy. Participants were recruited through Italian Red Cross branches working in settlements, which may introduce a selection bias. Communities maintaining established relationships with humanitarian organizations may differ from more isolated groups in terms of trust toward institutions, access to services, and health behaviors. In addition, systematic information on the number of individuals approached but declining participation was not consistently available across all participating branches, which limits the ability to estimate non-response bias. Missing responses were present for several variables, particularly among older participants and in relation to vaccination history. Analyses were therefore conducted using available data, and results should be interpreted with caution. Most variables were based on self-reported information, which may be affected by recall bias or social desirability bias. The survey design and sample size did not allow detailed subgroup analysis, which represents an important limitation of the study. In addition, the analyses relied primarily on descriptive and bivariate methods and did not include multivariable adjustment for potential confounding factors.

Another limitation concerns the scope of the gender analysis. While the study explored differences between male and female respondents across several social and health indicators, the survey did not include specific questions on reproductive health, early motherhood, or gender-based violence, which are recognized as important dimensions of vulnerability for Roma women. Future research should investigate these aspects in greater depth in order to provide a more comprehensive understanding of gender-related health inequalities within Roma and Sinti communities.

### 4.6 Implications for policy and practice

The findings of this study highlight the importance of addressing social determinants of health in Roma and Sinti communities. In particular, three priority areas emerge.

First, improving school attendance and educational attainment may have significant long-term health benefits, as education appears strongly associated with preventive health behaviors such as vaccination and screening uptake.

Second, interventions aimed at improving access to preventive healthcare services should consider cultural mediation and community-based outreach strategies, which may help overcome institutional mistrust and logistical barriers.

Third, policies addressing housing conditions and social inclusion remain essential to reduce structural inequalities affecting Roma and Sinti populations.

Public health programs designed in collaboration with community mediators and local organizations may represent an effective strategy to improve health literacy, vaccination coverage, and access to screening services within these communities.

## 5 Conclusion

Despite making up a small percentage of the population in Italy, the Roma and Sinti communities are greatly affected by social and economic exclusion, discrimination, and political disadvantages. Great effort by policymakers, healthcare workers and community members will be necessary to impact the wellbeing and growth of the Romani residing in Italy. Findings of this study suggest that sex, education and lifestyle changes could be highly impactful, although detailed studies of higher scope will be necessary in the future to establish clear relationships. Systemic discrimination and generational inequalities are difficult challenges to tackle, but the continued investigation and scrutiny of current systems can positively impact the outcomes of the Roma and Sinti communities, and ultimately improve the Italian quality of life as whole.

## Data Availability

The original contributions presented in the study are included in the article/Supplementary material, further inquiries can be directed to the corresponding author.
