# Supplementary material for: Health status and social determinants among Roma and Sinti communities in Italy: a cross-sectional study with a focus on sex-based inequalities
Source: Front Sociol. 2026 Apr 30;11:1717075. doi: 10.3389/fsoc.2026.1717075 (PMC13171302; doi:10.3389/fsoc.2026.1717075)
Supplement: Supplementary file 1 [file Supplementary_file_1.docx]

Supplementary Material

# Supplementary Data

|  | Good and very good | Fair | Bad o very bad |
| --- | --- | --- | --- |
| Low | 68,4 | 25,0 | 6,6 |
| Medium low | 60,9 | 31,4 | 7,7 |
| Medium high | 52,6 | 38,5 | 9,0 |
| High | 53,8 | 37,8 | 8,4 |
| χ2 Prob: 0.1174 |  |  |  |

*Tab 1. Persons by Housing distress and self-perceived heath status (percentage values)*

|  | No | Yes | Don’t know/Don’t remember |
| --- | --- | --- | --- |
| Low | 35,5 | 46,1 | 18,4 |
| Medium low | 52,7 | 19,6 | 27,7 |
| Medium high | 45,5 | 21,0 | 33,6 |
| High | 46,2 | 17,0 | 36,8 |
| χ2 Prob: <.0001 |  |  |  |

*Tab 2. Persons by Housing distress and influenza immunization (percentage values)*

|  | No | Yes | Don’t know/Don’t remember |
| --- | --- | --- | --- |
| Low | 15.9 | 73.8 | 10.3 |
| Medium low | 17.5 | 64.3 | 18.2 |
| Medium high | 17.0 | 61.4 | 21.6 |
| High | 19.2 | 58.2 | 22.0 |
| χ2 Prob: 0.0957 |  |  |  |

*Tab 3. Persons by Housing distress and COVID-19 vaccination (percentage values)*

|  | No | Yes | Don’t know/Don’t remember |
| --- | --- | --- | --- |
| Low | 39,0 | 47,5 | 13,6 |
| Medium low | 58,3 | 33,3 | 8,3 |
| Medium high | 75,6 | 17,9 | 6,4 |
| High | 58,9 | 32,1 | 8,9 |
| Pr<=P 0.0031 |  |  |  |

*Tab 4. Persons by Housing distress and Pap test in an asymptomatic patient (percentage values)*

|  | No | Yes | Don’t know/Don’t remember |
| --- | --- | --- | --- |
| Low | 56,5 | 21,7 | 21,7 |
| Medium low | 63,6 | 27,3 | 9,1 |
| Medium high | 81,3 | 12,5 | 6,3 |
| High | 33,3 | 46,7 | 20,0 |
| Pr<=P 0.0458 |  |  |  |

*Tab 5. Persons by Housing distress and mammogram in an asymptomatic patient (percentage values)*

# Supplementary Figures

*Figure 9. Cholesterol checks by gender (percentage values)*

*Figure 10. Cholesterol checks by nationality (%)*

*Figure 11. Blood glucose testing by gender (percentage values)*

*Figure 12. Blood glucose testing by nationality (percentage values)*

*Figure 13. Pap smear by nationality (percentage values)*

*Figure 14. Pap smear by school attendance (percentage values)*

*Figure 15. Mammography by age group (percentage values)*

*Figure 16. Mammography by school attendance (percentage values)*

*Figure 17. Mammography by nationality (percentage values)*

*Figure 18. Housing scarcity by gender (percentage values)*

*Figure 19. Housing scarcity by nationality (percentage values)*
